# Genome-wide identification of long non-coding RNAs reveals potential association with *Phytophthora infestans* asexual and sexual development

**DOI:** 10.1128/spectrum.01998-24

**Published:** 2025-03-26

**Authors:** Weilin Cao, Xiangming Pan, Ru Yu, Yuting Sheng, Hongxia Zhang

**Affiliations:** 1 The Engineering Research Institute of Agriculture and Forestry, Ludong University 12405 , Yantai, Shandong, China; 2 Shandong Provincial Key Laboratory of Biophysics, Institute of Biophysics, Dezhou University 71066 , Dezhou, Shandong, China; Barnard College, New York, USA

**Keywords:** long non-coding RNAs, *Phytophthora infestans*, asexual development, sexual reproduction

## Abstract

Long non-coding RNAs (lncRNAs) play pivotal roles in regulating diverse biological processes across plants, mammals, and fungi. However, the information on lncRNAs in oomycete asexual and sexual reproduction, which are two pivotal processes in the pathogenic cycle, has not been elucidated. In this present study, strand-specific RNA sequencing data of *Phytophthora infestans* with asexual development and sexual reproduction were reanalyzed, and a total of 4,399 lncRNAs were systematically identified. Compared to messenger RNAs (mRNAs), lncRNAs had a higher proportion of transcripts containing more than one exon, shorter nucleotide lengths, and lower expression levels. Target analysis showed that although only 280 lncRNA-mRNA pairs were shared, the functional pathways in which *cis* and *trans* targets participated were similar. Weighted gene co-expression network analysis of differentially expressed lncRNAs (DElncRs) and differentially expressed mRNAs (DEmRs) of asexual development stages indicated that lncRNAs might participate in different asexual stages and transformation of the growth stages via regulating functional genes. Expression trend analysis of DElncRs and DEmRs showed that lncRNAs may promote asexual development via upregulating mRNAs encoding development- and invasion-related proteins, such as INF6, triosephosphate isomerase, and glycoprotein elicitor. Co-expression analysis of DElncRs and DEmRs of sexual reproduction showed that lncRNAs could increase the level of mRNAs related to mating, such as M96 mating-specific protein and Crinkler family protein, which meant that lncRNAs might participate in sexual reproduction by regulating mating-related genes. Our study conducted a comprehensive analysis of lncRNAs in *P. infestans* and suggested a potential function of lncRNAs in asexual and sexual development.

**IMPORTANCE:**

This study systematically analyzed lncRNAs in *Phytophthora infestans*, revealing the associations between lncRNAs and functional genes. The potential regulatory roles of lncRNAs in the asexual and sexual reproduction stages were clarified, providing a new perspective for in-depth understanding of the reproductive regulatory network of oomycetes. This not only expands the understanding of the functions of non-coding RNAs in different biological groups but also provides potential targets for the development of new disease prevention and control strategies, promoting related research in the fields of agriculture and biology.

## INTRODUCTION

Oomycetes, as fungal-like microbes related to algae evolutionarily, are classified as stramenopile eukaryotes. They belong to the category of multicellular protists and possess distinctive genetic, physiological, and biological features (1). Among the oomycetes, multiple species, such as *Phytophthora*, *Pythium*, and downy mildew pathogens, can cause devastating diseases in hundreds of plant hosts, resulting in significant agricultural devastation and substantial economic losses (2, 3). The 3′-tag digital gene expression profiling technology was used to conduct a comprehensive analysis of the *Phytophthora sojae* transcriptome, revealing the differentially expressed genes and the regulatory networks at different growth stages (4). The sporangia (SP) stage can be further divided into two substages: sporangia and cleaving sporangia (CS). Additionally, cysts and germinating can be combined into a single stage called germinating cysts (GCs). The asexual development of *Phytophthora* is crucial for its attack and infection of plants, while the sexual stage contributes to its survival by generating new aggressive genotypes (5, 6). Oospores are produced by the pairing of homothallic (self-fertile) species and heterothallic (referred to as A1 and A2 in *Phytophthora*) of the two mating types (5). In addition, oospores can survive in the soil and maintain their infectivity for more than 36 months (7). However, information on the mating biology in oomycetes is rather limited. In *Phytophthora*, the regulation of functional genes by the identified mating hormones has been rarely reported (8–10). Previous studies have identified 87 mating-induced genes, but the incompleteness of the reference genome has limited its in-depth research (11, 12). Studies have shown that the M96 gene, which encodes a low-complexity extracellular protein, may be a component of oospore walls and is specifically expressed during the germination of sexual spores (13). Therefore, exploring the molecular mechanisms and characteristics underlying the oomycete asexual development and sexual reproduction could provide a theoretical basis to combat the diseases. To date, the molecular mechanisms associated with protein-coding genes of asexual and sexual cycles in oomycetes have been investigated by performing advanced high-throughput sequencing techniques (6, 14), whereas studies on non-coding transcripts are limited.

Non-coding RNAs (ncRNAs), as important players, can participate in the defense responses against virus, bacterium, and fungus attacks by regulating gene expression (15). Based on the sequence sizes, ncRNAs are divided into two types, including small ncRNAs (smRNAs) and long non-coding RNAs (lncRNAs) (16). The functions of smRNAs have been thoroughly studied based on several recent studies (17–19), whereas information on lncRNAs remains limited. The length of lncRNAs transcribed by RNA polymerase II (RNA Pol II) exceeds 200 nucleotides. This classification criterion based on length can distinguish them from small non-coding RNAs such as microRNAs and short interfering RNAs (20). According to the genomic location and transcription direction, lncRNAs can be further classed into four subclasses, including intergenic lncRNAs, intronic lncRNAs, antisense lncRNAs, and sense lncRNAs (21). LncRNAs play pivotal roles in various biological processes, including the modulation of protein activity and localization, the influence on other RNA molecules, and the generation of small or structural RNAs (22). Previous reports have revealed that there may be an association between lncRNAs in oomycetes and effector genes (23). These functions are achieved through *cis*- and *trans*-acting regulation mechanisms that target protein-coding genes (22, 24). Thus far, an lncRNA-like family in *Phytophthora infestans* has been previously described (25); however, there is a lack of genome-wide identification and functional studies on lncRNAs in this oomycete species.

In this study, the strand-specific RNA sequencing data of *P. infestans* with different asexual development stages and sexual reproductions were obtained and reanalyzed. The data set comprises asexual development stages, namely, mycelia (MY), SP, CS, zoospore (ZO), and GCs from two strains, 1306 and 88089. Additionally, it includes the sexual reproduction stage consisting of three different crosses. We identified 4,399 lncRNA candidates and found that the functional pathways involved in *cis*- and *tran*s-acting regulated targets are similar. We further constructed the lncRNA-messenger RNA (mRNA) networks that regulate asexual development and sexual reproduction, respectively. Our findings will provide a comprehensive analysis of lncRNAs in oomycetes and prove the potential association between lncRNAs and functional genes.

## MATERIALS AND METHODS

### Transcriptome data collection

The strand-specific RNA sequencing data of *P. infestans* was obtained from the National Center for Biotechnology Information (NCBI) (BioProject accession numbers PRJNA361417 and PRJNA445478) (12, 26) for identifying lncRNA. In order to verify the results, the RNA sequencing data of *P. infestans* related to mating (PRJNA369047) and at different growth times (PRJNA413149) were obtained from NCBI (27, 28).

### Bioinformatic pipeline for identification, annotation, and quantification of lncRNAs

The adaptors and low-quality bases in raw RNA-seq reads were filtered and removed using Trimmomatic (29). The remaining high-quality reads were mapped to *P. infestans* reference (https://protists.ensembl.org/Phytophthora_infestans/Info/Index?db=core) by performing the spliced read aligner HISAT (v.2.1.0) (30). The transcripts of each sample were assembled using StringTie software (v.2.1.3) (30). Subsequently, Gffcompare (v.0.11.2) was used to compare the assembled transcripts to the *P. infestans* genome annotation. The assembled transcripts were quantified using Salmon (v.0.14.1), and the transcript abundance was measured as mean transcript per million (TPM) (31).

A computational pipeline was designed to identify lncRNAs in *P. infestans*. First, the transcripts that were predicted to have an open reading frame of more than 100 amino acids using TransDecoder (https://github.com/TransDecoder/TransDecoder/releases) and a length of less than 200 bp were excluded. Then, the coding potential was calculated by performing CNCI (32), FLEK (33), and CPC2 (34). The transcripts labeled as coding in at least one of these software were removed. Subsequently, the remaining transcripts were compared against the UniProt (https://www.uniprot.org/) and Pfam protein (http://pfam.xfam.org) databases using BLASTP (E value of <0.001) in order to filter potential protein-coding genes.

### Target prediction

The protein-coding genes located within 10 kb 5′ upstream or 3′ downstream of lncRNA in the same scaffold are defined as potential *cis* targets (35). The expression levels were used to calculate the Pearson correlation between the protein-coding gene and lncRNA. The high correlation observed indicates a consistent expression pattern between lncRNA and gene across all samples, implying a potential shared role or regulatory relationship. Therefore, identifying genes that exhibit a high correlation as lncRNA targets serves as an optimal strategy for predicting the functional role of these lncRNAs. In this study, we considered protein-coding genes displaying a high Pearson correlation (|*r*| > 0.8 and *P* ≤ 0.05) as potential *trans* targets for the respective lncRNA.

### Differential abundance of lncRNAs and mRNAs

The differential expression analysis of lncRNAs and mRNAs was performed using the R package DESeq2 (v.1.38.3) based on read count data. Transcripts with a log_2_ (fold change) of ≥1 and a *P* value of ≤0.05 were considered significantly differentially expressed. The distribution of lncRNAs and mRNAs in all *P. infestans* scaffolds was drawn using TBtools (36). The R package ggplot was used to plot bubble charts, expression pattern maps, and heat maps. The R package TCseq2 was used to analyze expression trends of lncRNA and mRNA. TBtools software was used to plot the Venn diagram (36). Cytoscape software (v.3.10.0) was used to construct an interactive network between lncRNAs and targets (37). Kyoto Encyclopedia of Genes and Genomes (KEGG) enrichment analysis was performed to investigate the biological process of target genes using KOBAS (38).

### Weighted gene co-expression network analysis

The TPM of lncRNA and mRNA was used to construct a co-expression network by performing R package weighted gene co-expression network analysis (WGCNA) (39). The module-trait associations were estimated by calculating the correlation between the eigenvalues of lncRNA or mRNA modules and the developmental stages. The gene co-expression network in each module was a scale-free weighted gene network containing nodes and edges. The satisfactory soft power threshold was determined to be 19. The adjacency function was employed to construct adjacency matrices in accordance with the soft power threshold. Based on the adjacency matrix, the topological overlap (TO) matrix similarity algorithm was used to generate TOM, which was then employed in the hierarchical clustering of lncRNAs or mRNAs. A hierarchical clustering dendrogram was obtained by adopting the Dynamic tree-cutting algorithm. Further, the average TO values of all lncRNAs and mRNAs in modules were compared, and any modules with lower TO values were excluded. Modules with a correlation coefficient *r* of >0.7 were considered highly correlated. A positive correlation represents higher/preferential expression of lncRNAs and mRNAs in a particular stage compared to all the other stages.

## RESULTS

### Genome-wide identification of *P. infestans* lncRNAs

**Fig 1:**
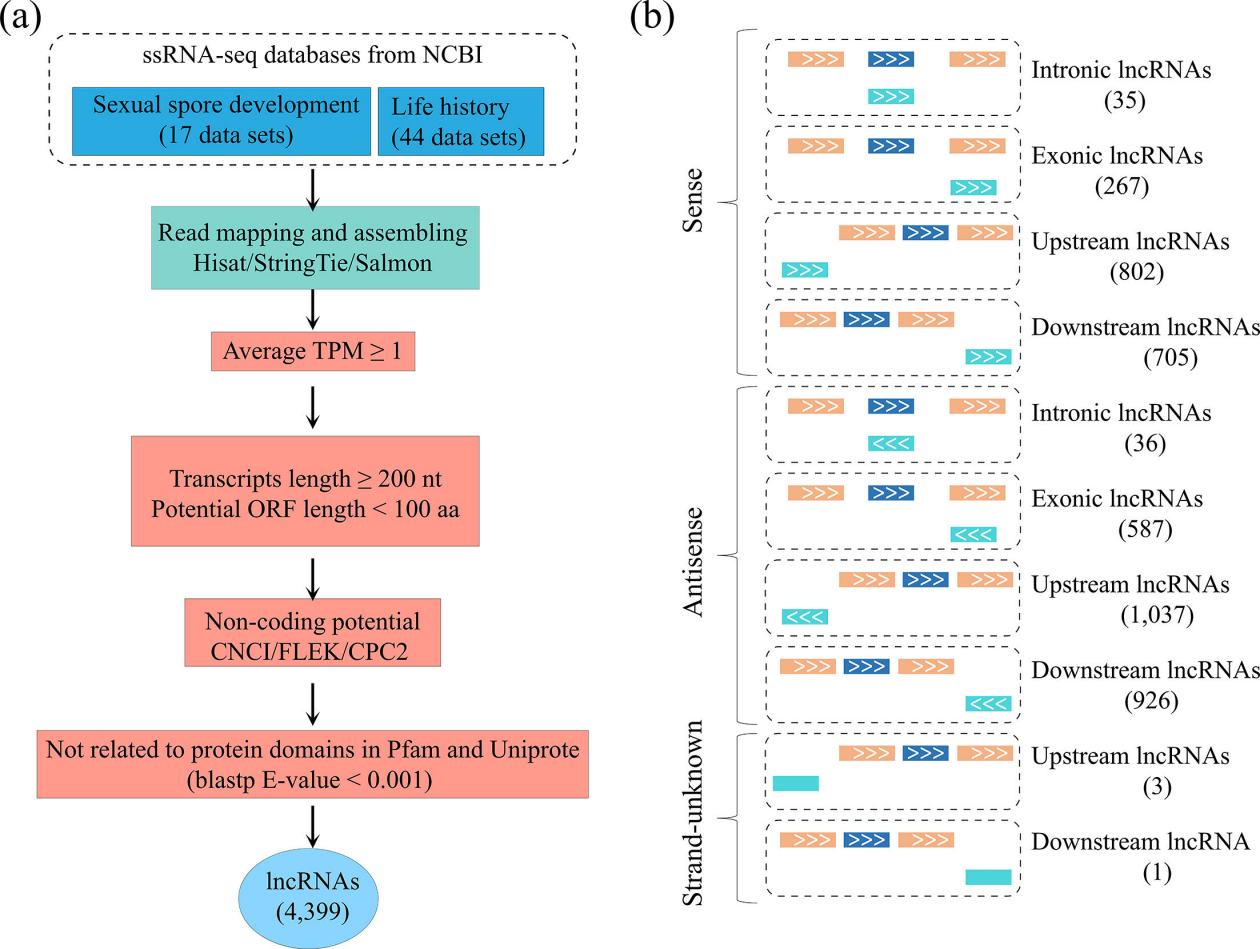
Pipeline of *Phytophthora infestans* long non-coding RNA (lncRNA) identification. (a) The computational pipeline for identifying *P. infestans* lncRNAs from strand-specific RNA sequencing data. (b) Diagram and abundance of different types of lncRNAs. The upper line in each box represents a target gene; the lower line is the lncRNA; the blue boxes are introns. aa, amino acid; CNCI, coding-non-coding index; CPC2, Coding Potential Calculator 2; ORF, open reading frame; TPM, transcripts per million.

To investigate comprehensive information of *P. infestans* lncRNAs, high-throughput strand-specific RNA-seq data in 61 *P*. *infestans* samples, including asexual development (44 data sets) and sexual reproduction (17 data sets) from the NCBI database, was obtained and reanalyzed (Table S1). A total of 3.02 billion reads were generated, and approximately 45.69% were mapped to the *P. infestans* genome. A total of 80,265 assembled transcripts were used to systematically identify *P. infestans* lncRNAs via a computational pipeline (Fig. 1a). First, a total of 11,608 mRNAs were identified and removed by comparing with the available gene annotation data from the reference genome database (https://protists.ensembl.org/Phytophthora_infestans/Info/Index/). Subsequently, the remaining transcripts with an average expression-level TPM of more than one and a sequence length of more than 200 nucleotides were used to calculate protein-coding potential via five approaches, including CNCI, FLEK, CPC2, Pfam, and Uniprote. Ultimately, 4,399 transcripts as putative lncRNAs were obtained (Fig. 1a). According to the direction between the lncRNA and the closest protein-coding gene in the same scaffold, three types of lncRNA were classified, including sense lncRNA (1,809) with the same transcription direction, antisense lncRNA (2,586) with the opposite transcription direction, and strand unknown lncRNA (4) (Fig. 1b; Table S2). Each type of lncRNA was further divided into four subtypes including intronic, exonic, upstream, and downstream lncRNAs. There were 35 intronic, 267 exonic, 802 upstream, and 705 downstream lncRNAs from sense lncRNAs and 36 intronic, 587 exonic, 1,037 upstream, and 926 downstream lncRNAs from antisense lncRNAs (Fig. 1b).

### Characteristics of the identified *P. infestans* lncRNA

**Fig 2:**
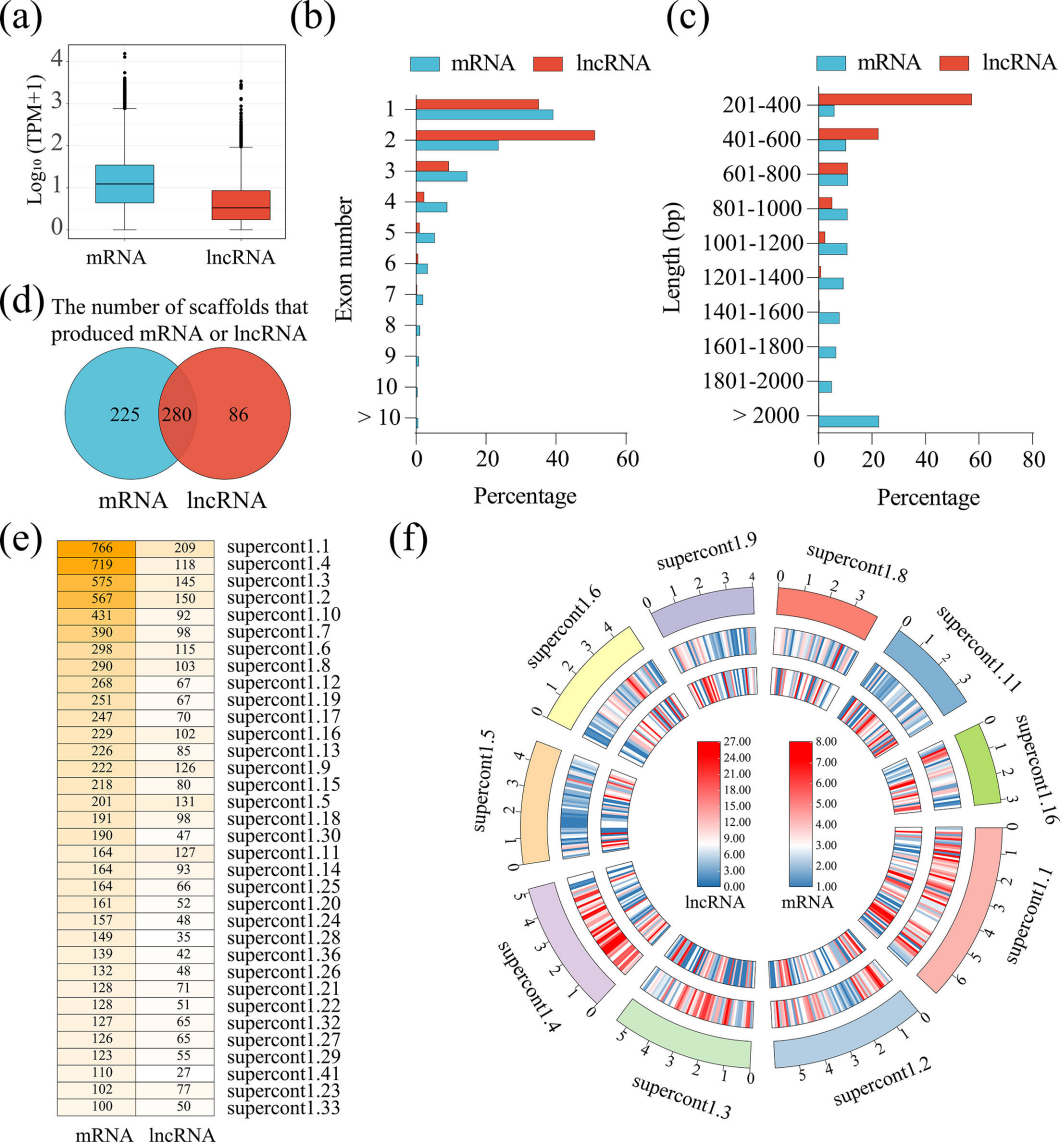
Characterization of *P. infestans* lncRNAs. (a) The expression levels of lncRNAs and mRNAs in all samples. (b) Percentage of lncRNAs and mRNAs containing different numbers of exons. (c) Percentage of lncRNAs and mRNAs with different lengths. (d) Venn diagram showed the number of scaffolds producing lncRNA and mRNA. (e) Heatmap shows the scaffolds producing more than 100 lncRNAs or mRNAs. (f) Distribution of lncRNAs and mRNA along *P. infestans* scaffolds producing more than 100 mRNAs and lncRNAs. From the outside to the inside, there are chromosomes, mRNA, and lncRNA.

The expression level of each transcript was estimated, and the results indicated that the median levels of lncRNAs (average TPM of all samples) were significantly lower than that of mRNAs (*P* value < 0.001) (Fig. 2a; Table S3). The proportion of lncRNAs with multiple exons was 63.76%, indicating that these lncRNAs underwent splicing, which was higher than the 60.78% observed in mRNAs (Fig. 2b). Additionally, the lengths of *P. infestan*s lncRNAs were significantly shorter than those of the mRNAs (median length: 358 bp vs 1,227 bp) (Fig. 2c). According to the reference genome, *P. infestan*s contains 4,921 scaffolds, in which 505 and 366 scaffolds were found to generate mRNAs and lncRNAs, respectively, and 280 shared scaffolds produced both transcripts (Fig. 2d). In the shared scaffolds, only 34 scaffolds generated more than 100 mRNAs, with 10 of them generating over 100 lncRNAs (Fig. 2e). Subsequently, the distribution of the mRNAs and lncRNAs was examined across the 10 scaffolds that generated over 100 transcripts for both mRNAs and lncRNAs, revealing an even distribution pattern (Fig. 2f). The conservation of *P. infestan*s and *P. sojae* lncRNA sequences was analyzed by performing BLASTN searches (*E* value = 1 × 10^−5^), and the results showed that only 31 *P*. *infestan*s lncRNAs had hits in *P. sojae* lncRNAs, indicating the less conservation of *P. infestan*s lncRNAs (Table S4).

### Predicting targets of *P. infestans* lncRNAs

**Fig 3:**
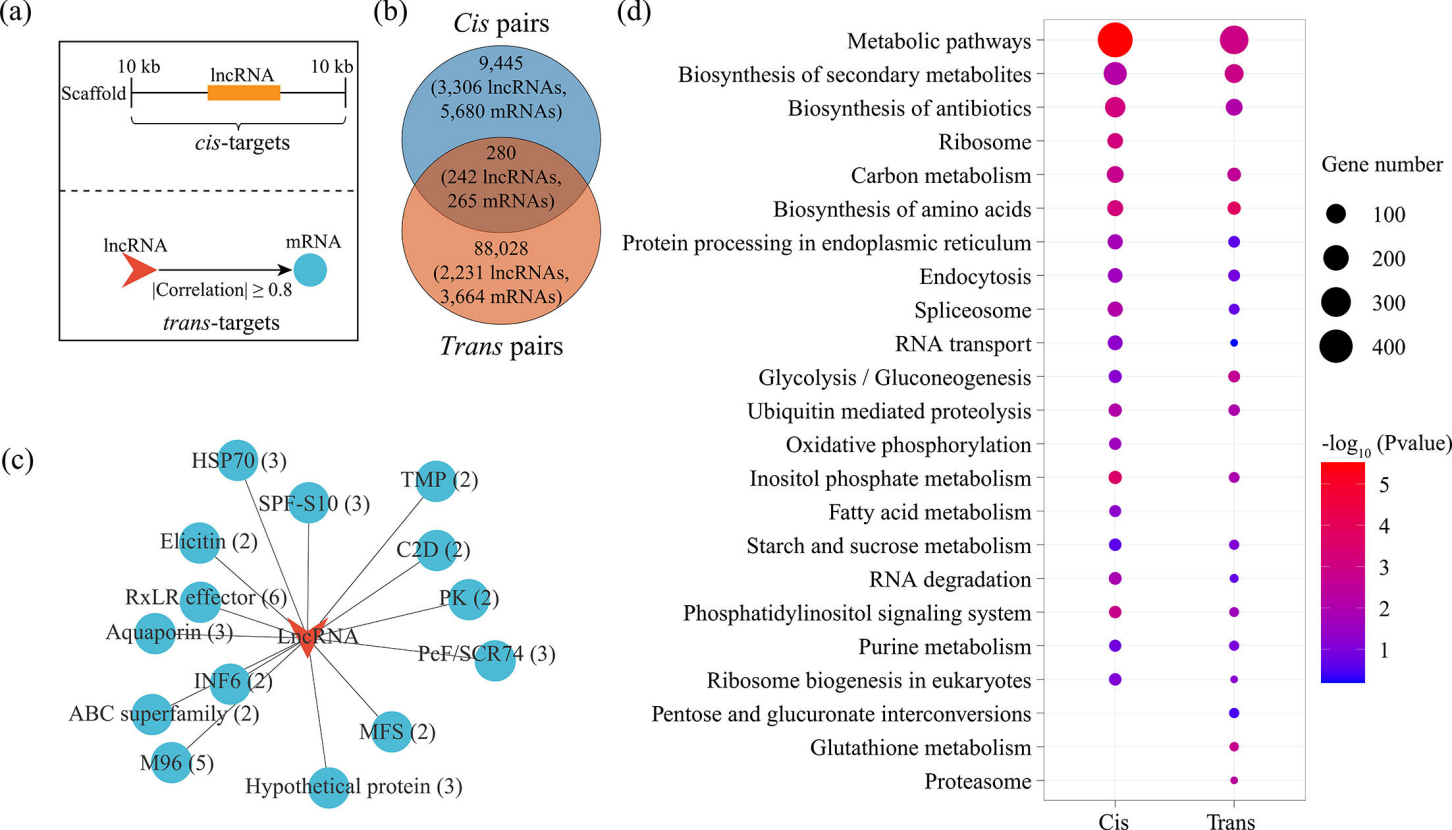
Prediction and characterization of *P. infestans* lncRNA targets. (a) The methods of predicting *cis* and *trans* targets. (b) Venn diagram shows the number of *cis* and *trans* pairs. (c) Construction of lncRNA-mRNA network in shared pairs between *cis* and *trans* pairs. (d) KEGG enrichment analysis of *cis* and *trans* targets and the top pathways ranked by gene number. The size of the circles represents the gene number. Blue to red circles represent *P* values from low to high.

Since lncRNAs play important roles in the biological process by regulating target gene expression, the interaction pairs of lncRNA and mRNA were predicted based on location (*cis*, ≤10 kb) and expression patterns (*trans*, |correlation| ≥ 0.8, *P* value of <0.05), respectively (Fig. 3a; Table S5). The results revealed the identification of 9,725 *cis* (5,818 mRNAs) and 88,308 *trans* (3,686 mRNAs) pairs and a mere 280 shared pairs (266 mRNAs) (Fig. 3b; Table S5). In the shared pairs, multiple mRNA encoding invasion and mating-related proteins were targeted, such as secreted RxLR effector peptide protein (six mRNAs) and M96 mating-specific protein (five mRNAs) (Fig. 3c). In addition, KEGG enrichment analysis of mRNAs from *cis* and *trans* pairs was performed, respectively, resulting in the identification of 17 shared pathways of the top 20 pathways ranked by mRNA number, including carbon metabolism, endocytosis, and ubiquitin-mediated proteolysis (Fig. 3d). This suggests that despite a lower number of shared pairs, the biological processes regulated by lncRNAs through *cis*- and *trans*-acting regulation patterns were similar.

### Functional analysis of asexual development stage-related lncRNAs

**Fig 4:**
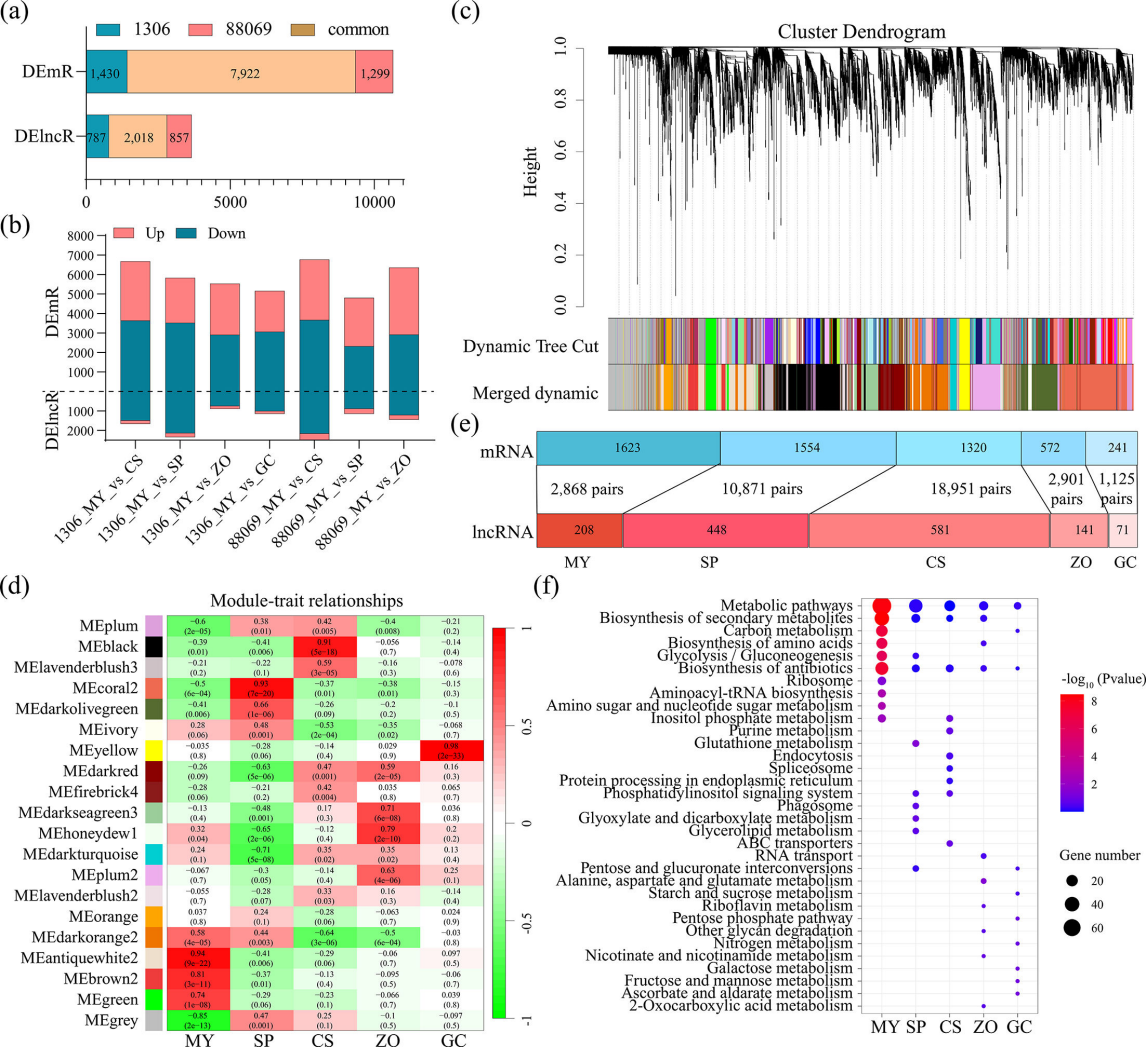
Identification and characterization of asexual development stage-related lncRNAs. (a) The number of all differentially expressed lncRNAs (DElncRs) and differentially expressed mRNAs (DEmRs) in strains 1306 and 88069. (b) The distribution of DElncRs and DEmRs in strains 1306 and 88069. (c) Hierarchical clustering tree (dendrogram) of the lncRNAs and mRNAs based on co-expression network analysis. Each short vertical line (leaf) represents an individual transcript. Branches represent the modules containing highly interconnected genes. Different colors of the dynamic tree represent different modules. The similar modules were combined. (d) Module-trait relationships. Each row represents a module eigen transcript (correlation between a column and a trait). The correlation and *P* value in each cell are shown. Green to red cells represent the correlation from low to high. (e) The number of lncRNA-mRNA pairs, along with the lncRNAs and mRNAs contained in modules, highly correlated with different asexual development stages. The number of lncRNA-mRNA pairs in the modules that are highly correlated with different asexual development stages. (f) KEGG enrichment analysis of mRNAs from lncRNA-mRNA pairs in highly correlated modules. The size of the circles represents the gene number. Blue to red circles represent *P* values from low to high.

To further explore the roles of lncRNA during development stages, we identified the differentially expressed lncRNA (DElncR) and differentially expressed mRNA (DEmR) of SP, CS, ZO, and GCs, compared to MY in strains 1306 and 88069, respectively. We obtained a total of 3,662 DElncRs and 10,651 DEmRs from all comparison groups with an absolute fold change of ≥2 and a *P* value of <0.05 (Fig. 4a and b; Table S6). All of the DElncRs and DEmRs were used to comprehensively identify development stage-related modules by performing WGCNA. After constructing the transcript cluster scheme power value (*β* value, 8), transcripts exhibiting similar expression patterns within the same subnetworks were allocated to corresponding co-expression modules (Fig. 4c). Finally, 20 modules with correlation ranging from −0.85 to 0.98 were obtained (Fig. 4d). Among the modules, eight modules were significantly correlated (*P* value of <0.05, *r* > 0.7) with different stages. For instance, the antiquewhite2 module showed a strong correlation (*r* = 0.94) with mycelia, while brown2 (*r* = 0.81) and green modules (*r* = 0.74) were significantly correlated with mycelia as well. The coral2 module displayed a high correlation coefficient (*r* = 0.93) with sporangia, whereas the black module exhibited a strong correlation (*r* = 0.91) with cleaving sporangia. Additionally, darkseagreen3 (*r* = 0.71) and honeydew1 (*r* = 0.79) demonstrated significant correlations with zoospores, whereas yellow modules (*r* = 0.98) were highly related with germinating cysts (Fig. 4d). These highly correlated modules contained 1,449 lncRNAs, and the characteristic analysis revealed that 51.03% of them contained two exons and only 5.34% were longer than 1,000 bp (Fig. S1). In the highly correlated modules, there were 2,868, 10,871, 18,951, 2,901, and 1,125 DElncR-DEmR pairs involved in MY, SP, CS, ZO, and GC, respectively (Fig. 4e), suggesting that the DElncRs may play important roles in different stages via regulating targets. To explore the biological processes regulated by DElncR-targeted DEmRs, all of the DEmRs from *cis* and *trans* DElncR-DEmR pairs in highly correlated modules were further used to perform KEGG pathway enrichment analysis (Fig. 4f). The results showed that multiple pathways were involved in one or more development stages. For instance, the biosynthesis of antibiotics was observed throughout all stages, while the biosynthesis of amino acids occurred in two stages (MY and ZO). Additionally, inositol phosphate metabolism was identified in two stages (MY and CS); phosphatidylinositol signaling system was present in two stages (SP and CS); and phagosome activity was detected specifically in SP (Fig. 4f). The aforementioned findings collectively indicated that lncRNAs function as regulatory factors in different development stages by regulating mRNA associated with multiple biological pathways.

### Identification of lncRNAs related to stage transitions

**Fig 5:**
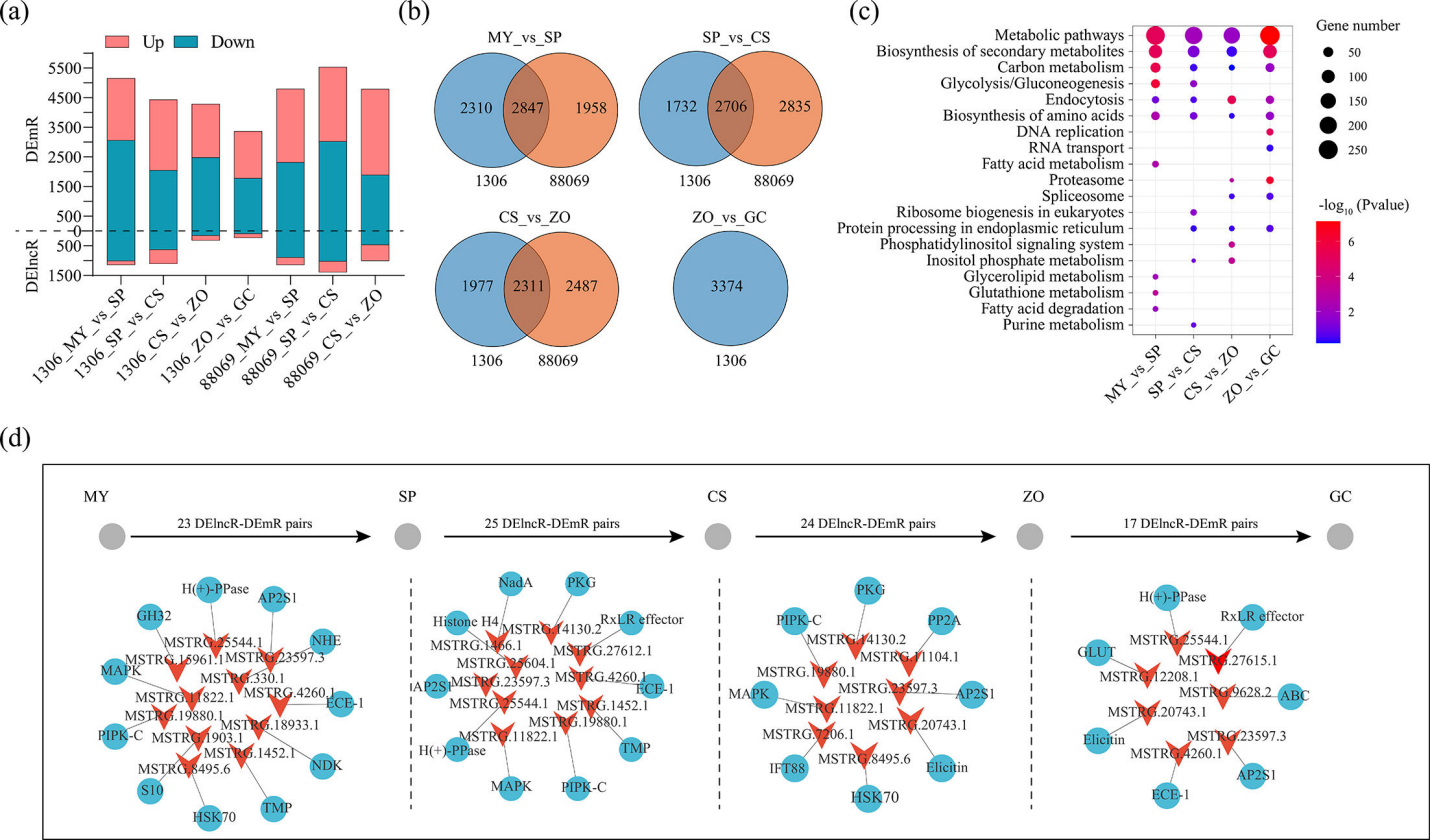
Identification of lncRNAs related to stage transitions. (a) The number of all differentially expressed lncRNAs (DElncRs) and differentially expressed mRNAs (DEmRs) between each stage and its preceding stage. (b) The Venn diagram shows the distribution of DEmRs in different comparisons between strains 1306 and 8806. (c) KEGG enrichment analysis of the shared DEmRs between 1306 and 8806. The size of the circles represents the gene number. Blue to red circles represent the *P* values from low to high. (d) The key lncRNA-mRNA pairs of each transformation process of *P. infestans*.

To explore the key factors in the process of stage transition, we identified the DElncRs and DEmRs between each stage and its preceding stage. (Fig. 5a; Table S7). Between strains 1306 and 8806, 2,847, 2,706, and 2,311 shared DEmRs in comparisons of MY vs SP, SP vs CS, and CS vs ZO were identified, respectively (Fig. 5b). In addition, a total of 3,374 DEmRs were identified in the comparison of ZO vs GC (Fig. 5b). KEGG enrichment analysis of the shared DEmRs has shown that metabolic pathways, biosynthesis of secondary metabolites, endocytosis, and biosynthesis of amino acids were significantly enriched during all stages (Fig. 5c). The shared DElncR-DEmR pairs between strains 1306 and 8806 were identified in each transition in the growth stages (Fig. 5d). There were 23, 25, and 24 pairs identified, respectively, in the transitions from MY to SP, from SP to CS, and from CS to ZO. For the transition from ZO to GC, a total of 17 pairs were identified (Fig. 5d).

### Construction of the lncRNA-mRNA network during *P. infestans* asexual development

**Fig 6:**
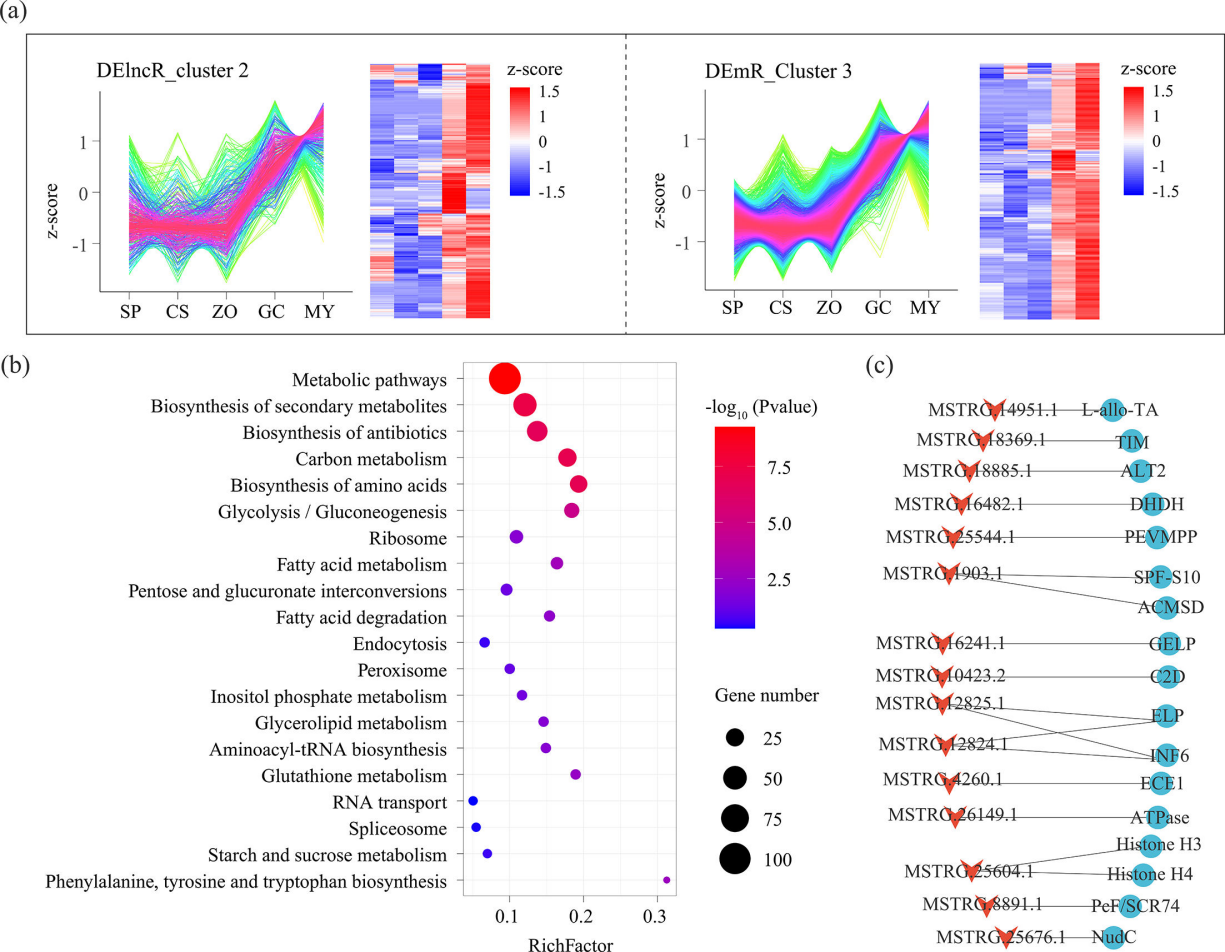
Express trend analysis of all DElncRs and DEmRs from asexual development samples. (a) R package TCseq was used to cluster DElncRs and DEmRs from asexual development samples, and the continually upregulated DElncRs and DEmRs are shown. (b) KEGG enrichment analysis of DEmRs from lncRNA-mRNA *cis* and *trans* pairs generated by DElncR_cluster2 and DEmR_cluster3. The size of the circles represents the *P* value. Blue to red circles represent the gene number from low to high. (c) Construction of lncRNA-mRNA networks using lncRNAs and mRNAs from shared pairs between *cis* and *trans* patterns.

To identify lncRNA-mNRA regulated networks promoting asexual development, the expression trends of DElncRs and DEmRs crossing all development stages were analyzed, respectively, by performing R package TCseq. Based on expression patterns, DElncRs and DEmRs were clustered into six types, including DElncR_clusters 1–6 and DEmR_clusters 1–6, respectively. The expression levels of 426 DElncRs and 2,451 DEmRs from DElncR_cluster 2 and DEmR_cluster 3 were continuously elevated during development (Fig. 6a). Among them, 254 *cis* and 5,044 *trans* pairs were obtained, including 265 DElncRs and 871 DEmRs. Subsequently, KEGG enrichment analysis of the DEmRs was performed, and the results showed that multiple targeted DEmRs related to development and invasion pathways were regulated by DElncRs, such as biosynthesis of antibiotics, carbon metabolism, and inositol phosphate metabolism (Fig. 6b). Furthermore, a total of 47 shared pairs between *cis* and *trans* pairs were observed, including 36 DElncRs and 45 DEmRs. Among them, the DEmRs encoding development- and invasion-related proteins, such as INF6, triosephosphate isomerase, and glycoprotein elicitor, were positively regulated by DElncRs (Fig. 6c). To verify the results, we analyzed the expression patterns of 10 lncRNAs from DElncR_cluster 2 using RNA sequencing data related to *P. infestans* growth. The results showed that the expression patterns of these 10 genes in the two data sets were similar, indicating that the results were reliable (Fig. S2a).

### Identification and functional analysis of mating-induced lncRNAs

**Fig 7:**
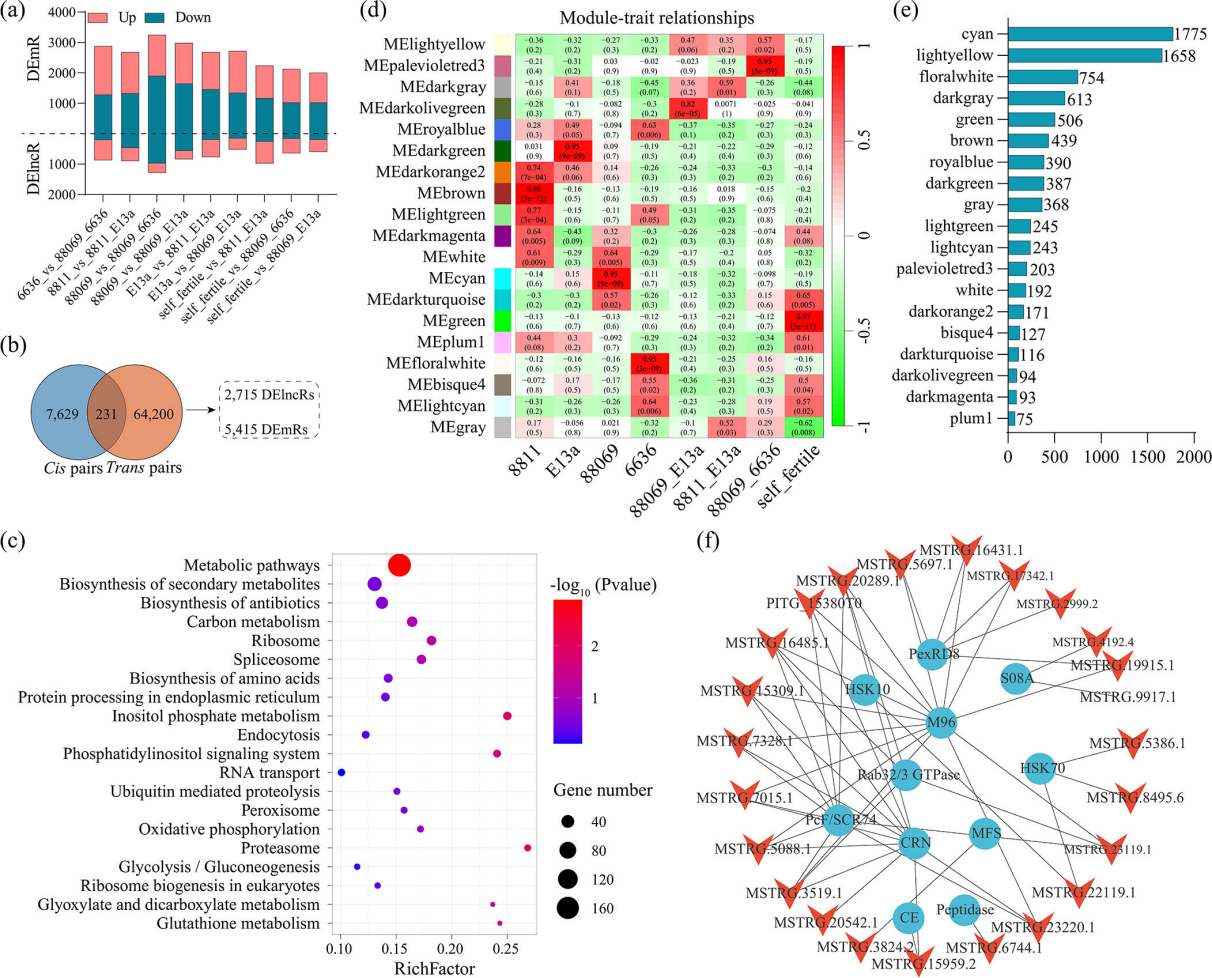
Identification of mating-induced lncRNAs. (a) The distribution of DElncRs and DEmRs in mating compared to non-mating and self-fertile strains. (b) Venn diagram shows the number of cis and *trans* pairs and the lncRNA and mRNA contained. (c) KEGG enrichment analysis of DEmRs from *cis* and *trans* pairs in sexual reproduction. (d) Module-trait relationships. Each row represents a module eigen transcript (correlation between a column and a trait). The correlation and *P* value in each cell are shown. Green to red cells represent the correlation from low to high. (e) The number of transcripts including lncRNAs and mRNAs in each module. (f) Construction of networks using lncRNA-mRNA pairs from highly correlated modules with mating.

To investigate the functional roles of lncRNAs in *P. infestans* sexual reproduction, the RNA-seq data of four non-mating (A1: 8811 and 88069, A2: 6636 and E13a), three mating (A1 plus A2: 88069_E13a, 8811 × E13 a, and 88069 × E13 a), and one self-fertile strain were reanalyzed. The differential expression analysis revealed that, compared to non-mating (parental lines) and self-fertile strains, a total of 3,563 DElncRs and 11,232 DEmRs were identified in the mating strain based on the criteria of an absolute fold change of ≥2 and a *P* value of <0.05 (Fig. 7a; Table S8). Among them, 7,860 *cis* and 64,431 *trans* pairs were identified, including 2,715 DElncRs and 5,415 DEmRs (Fig. 7b). Then, the DEmRs were subjected to KEGG pathway enrichment analysis, and multiple sexual reproduction-related pathways were enriched, including biosynthesis of antibiotics (35 targets), endoplasmic reticulum (16 targets), and phosphatidylinositol signaling system (13 targets) (Fig. 7c). To further explore the regulatory network of sexual development, the WGCNA was performed using DElncRs and DEmRs induced by mating. Finally, a total of 19 modules with transcript numbers of 75–1,775 were obtained (Fig. 7d and e), in which 2 modules were highly correlated (*r* > 0.7 and *P* value of <0.05) with 2 mating, including the darkolivegreen module with 88069_E13a (*r* = 0.82) and the palevioletred3 module with 88069 × 6636 (*r* = 0.95) (Fig. 7d). These two modules contain 27 lncRNAs, among which 15 lncRNAs contain two exons, and only 9 lncRNAs were more than 500 bp in length. In the highly correlated modules, 5 *cis* and 33 *trans* pairs in darkolivegreen module and 4 *cis* and 99 *trans* pairs in palevioletred3 module were obtained. In the DElncR-DEmR pairs from the highly correlated modules, multiple regulatory networks were associated with sexual reproduction, such as 14 DElncRs targeting PITG_05896 and PITG_22785 encoding M96 mating-specific protein and 11 DElncRs targeting PITG_18145 and PITG_04751 encoding the Crinkler (CRN) family protein (Fig. 7f). The results suggested that *P. infestans* lncRNAs participate in sexual reproduction via regulating targets related to mating. To further validate the reliability of the research results, we carefully selected 10 lncRNAs that were significantly upregulated during the mating process and conducted an analysis of their expression patterns during the mating of other *P. infestans* strains. This similar expression pattern confirmed the reliability of the results (Fig. S2B).

## DISCUSSION

Previous reports have proven that lncRNAs play vital roles in regulating the expression of genes related to asexual development and sexual reproduction in plants or animals (40–45). However, the available data on *P. infestans* lncRNA regulation of asexual development and sexual reproduction were limited. In this work, a total of 4,399 lncRNA candidates in *P. infestans* were identified by reanalyzing strand-specific RNA-seq data from the NCBI database. Consistent with previous research, the transcript length and transcription level of lncRNAs were also shorter and lower than mRNAs in *P. infestans* (23). In addition, *cis* and *trans* targets were predicted based on the genomic location and transcription pattern, and the biological processes involved in mRNAs from *cis* and *trans* pairs were similar, suggesting that both *cis* and *trans* are reliable ways to predict targets of lncRNAs. As the first comprehensive identification of lncRNAs in *P. infestans*, our research provided the basis for further studying functional roles of lncRNAs in asexual development and sexual reproduction of oomycetes.

During asexual development, multiple DElncR-DEmR pairs were highly correlated with different stages. A total of 601 DEmRs highly correlated with mycelia were identified from 2,868 pairs, among which 7 DEmRs were found to be involved in the inositol phosphate metabolism pathway, and 6 DEmRs were associated with the amino sugar and nucleotide sugar metabolism pathway. The inositol phosphate system orchestrates nutrient sensing, growth factor signaling, and cellular energy storage via regulating mTOR signaling, AMPK, AKT, and energy-sensing pathways (46). Amino sugars play a crucial role as essential constituents in microbial secondary metabolism (47). Therefore, lncRNAs may regulate mycelia growth by targeting the functional genes. For sporangia, the DElncR targeted DEmRs were associated with glutathione metabolism (four DEmRs), glyoxylate and dicarboxylate metabolism (three DEmRs), and glyoxylate and dicarboxylate metabolism (three DEmRs). The pathways may remain metabolically active in sporangia to store energy for cytoplasmic cleavage and the subsequent release of zoospores (48). During cleaving sporangia, the DEmRs from DElncR-DEmR pairs in highly correlated modules were involved in metabolic pathways, such as purine metabolism, protein processing in endoplasmic reticulum, and inositol phosphate metabolism, which may play a pivotal role in facilitating the significant morphological changes. Three mRNAs from DElncR-DEmR pairs of zoospore-related modules encode a drug/metabolite transporter, implying the lncRNAs may improve the infection capacity of *P. infestans*. For germinating cysts, there were only nine functional genes from the DElncR-DEmR pairs identified, suggesting that the roles of lncRNAs in this stage need to be further explored. In addition, 5,251 DElncR-DEmR pairs containing 265 DElncRs and 871 DEmRs showed sustained increased expression trends crossing whole developmental stages, indicating the DElncRs might be time course-dependent regulators to promote development by targeting DEmRs. These DEmRs were mainly enriched in carbon metabolism (26 DEmRs), biosynthesis of amino acids (23 DEmRs), and glycolysis/gluconeogenesis (16 DEmRs) pathways, suggesting that DElncR may regulate *P. infestans* asexual development by targeting metabolism-related mRNAs to facilitate energy production and nutrient acquisition. Multiple mRNAs that were significantly differentially expressed during the growth stage transition of *P. infestans* may be regulated by lncRNA. For example, from MY to SP and from CS to ZO, the DEmR PITG_12186 encoding sporangia induced mitogen-activated protein kinase was regulated by DElncR MSTRG.11822.1. In *Phytophthora sojae*, PsMPK1, as an SLT2-type mitogen-activated protein kinase, plays a crucial role in zoosporogenesis, hyphal growth, pathogenicity, and cell wall integrity (49). During the sporulation stage and biotrophic stage when *P. infestans* infects plants, nitrosative stress enhanced the total acetylation of histone H3/H4 and some histone acetylation marks (50). In this study, we found that lncRNA MSTRG.25604.1 and its target PITG_05676 were differentially expressed during the transformation stage from SP to CS. These results suggested that these DElncR-DEmR pairs may play a crucial role in the transformation of the growth stages of *P. infestans*.

Compared to asexual development, the molecular aspects of sexual development in oomycetes were considerably more limited. In *Phytophthora*, the genes regulating oospore formation were identified (51, 52), and the oomycete *Pythium oligandrum* protein that decorates the oospore surface has been proven (53). However, as of now, the roles of lncRNAs in sexual spores remain unexplored. Thus, this study aims to uncover novel mechanisms underlying the lncRNA-mRNA interaction in the induction of mating. The lncRNA-mRNA regulatory networks constructed during mating revealed the presence of numerous genes involved in mating induction, including heat shock 70 kDa protein (HSP70) and M96 mating-specific protein (M96). The HSP70 gene plays a pivotal role in regulating reproduction in *Ophraella communa*, and it has the ability to stimulate male mating behavior (54). M96, as a sexual sporogenesis-specific induced gene, encodes extracellular protein that may promote formation of oospore walls (13). Therefore, the lncRNA-mRNA networks may be contributors for the formation of oospore walls or the synthesis of extracellular substances that bind antheridia to oogonia during mating. Surprisingly, three and two genes belonging to the CRN and RxLR protein families were regulated by 11 and 5 lncRNAs, respectively. It has been consistently demonstrated that CRN and RxLR proteins always play roles in suppressing plant immunity during infection. According to this work, in addition to being related to immunity, CRN and RxLR proteins may also play important roles in mating. In addition, there were six shared DElncR-DEmR pairs that were closely related to both mating and asexual development, including three DElncRs and four DEmRs. Among the DEmRs, PITG_04751 encoding CRN and PITG_02902 encoding carbohydrate esterase were observed, suggesting that the DElncRs may be involved in both sexual and asexual development by regulating the functional mRNAs.

The transcriptomic dynamics of some oomycetes such as *Phytophthora sojae* and *Phytophthora parasitica* have been reported (4, 55). However, these studies primarily focused on the functional roles of genes, neglecting the characterization of lncRNAs. The present study conducted a comprehensive transcriptomic analysis at both the asexual and sexual stages, thereby enhancing the scale of investigation. We found that lncRNAs were associated with mating and asexual development stages in *P. infestans* via regulating mRNAs, suggesting their potential functions in sexual and asexual development. However, further investigations are required to validate the regulatory network and elucidate the functional mechanisms underlying lncRNA-mediated regulation.

## Data Availability

All of the data sets used in this study can be obtained from online repositories. The RNA-seq data and accession numbers can be found in the article.

## SUPPLEMENTAL MATERIAL

Supplemental figures spectrum.01998-24-s0001.docx Fig. S1 and S2.

Table S1 spectrum.01998-24-s0002.xlsx The details of all samples obtained from NCBI.

Table S2 spectrum.01998-24-s0003.xlsx Identification of *P. infestans* lncRNAs.

Table S3 spectrum.01998-24-s0004.xlsx Transcripts per million of lncRNAs and mRNAs in each sample.

Table S4 spectrum.01998-24-s0005.xlsx Conservative analysis of *P. infestans* lncRNAs.

Table S5 spectrum.01998-24-s0006.xlsx Prediction of lncRNA targets.

Table S6 spectrum.01998-24-s0007.xlsx Identification of differentially expressed lncRNAs and mRNAs in asexual development stages.

Table S7 spectrum.01998-24-s0008.xlsx Differentially expressed lncRNAs and mRNAs between each growth stage and its preceding stage.

Table S8 spectrum.01998-24-s0009.xlsx Identification of differentially expressed lncRNAs and mRNAs in sexual reproduction.

ASM does not own the copyrights to Supplemental Material that may be linked to, or accessed through, an article. The authors have granted ASM a non-exclusive, world-wide license to publish the Supplemental Material files. Please contact the corresponding author directly for reuse.
