# Supplementary material for: Genome-wide identification of long non-coding RNAs reveals potential association with Phytophthora infestans asexual and sexual development
Source: Microbiol Spectr. 2025 Mar 26;13(5):e01998-24. doi: 10.1128/spectrum.01998-24 (PMC12054190; doi:10.1128/spectrum.01998-24)
Supplement: Supplemental figures — Fig. S1 and S2. [file spectrum.01998-24-s0001.docx]

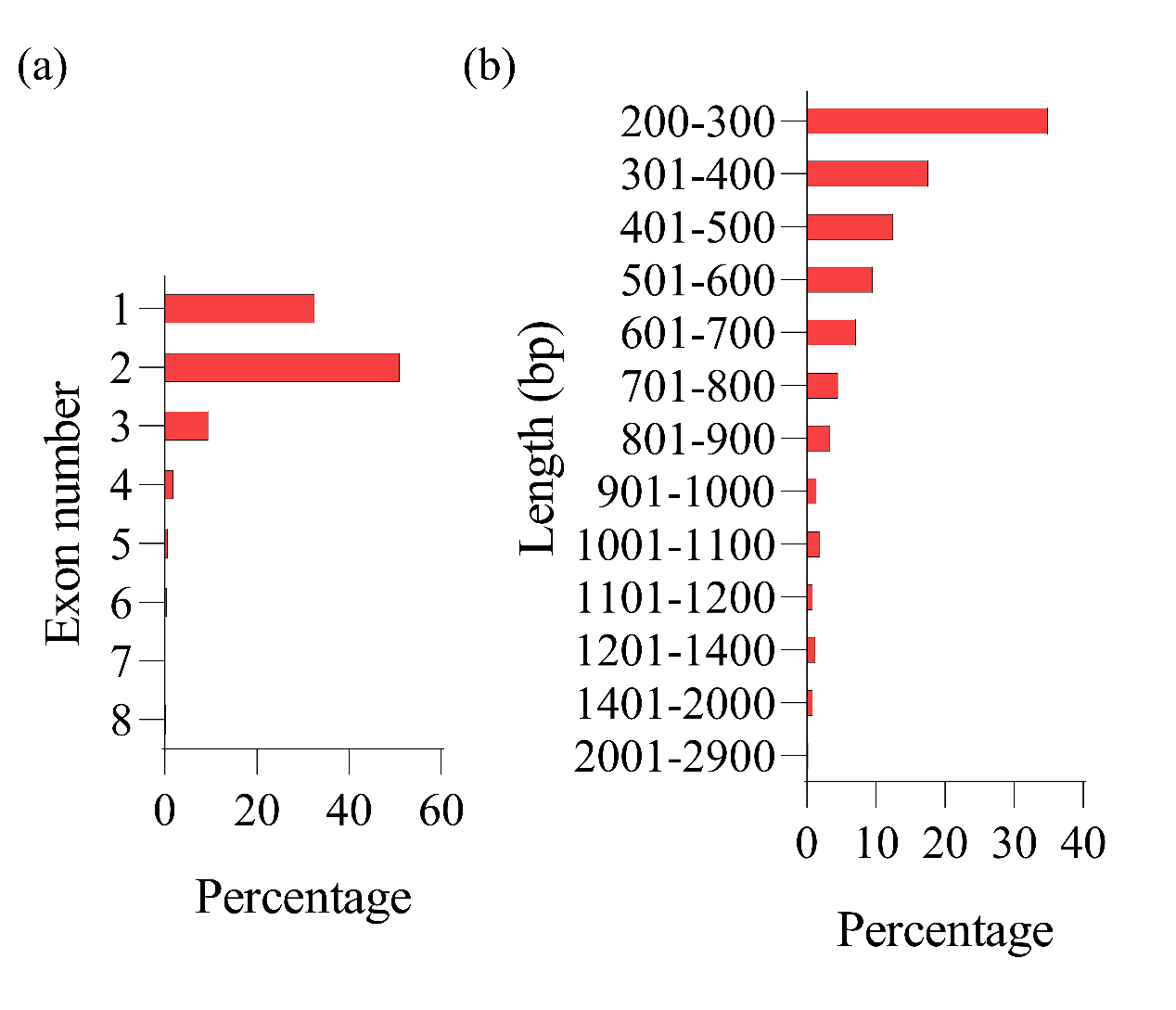


**FIG S1.** Characteristics of the lncRNAs related to asexual development of P*. infestans*.


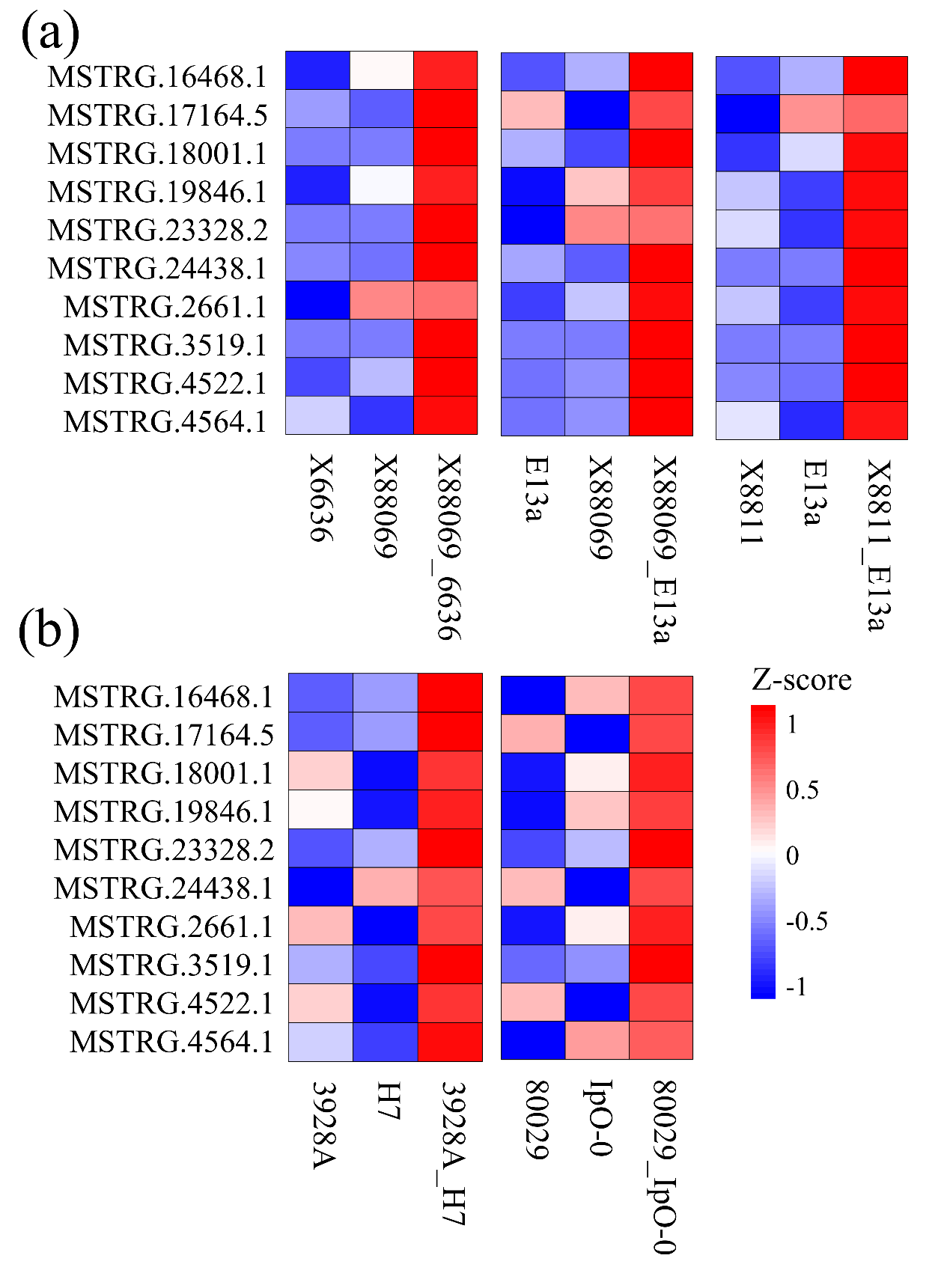


**FIG S2.** Characteristics of the lncRNAs related to asexual development of P. infestans.
